# Pan-Genomic Analysis and Functional Characterization of the *ATXR* Gene Family Highlights Its Role in Regulating Agronomic Traits in Rapeseed

**DOI:** 10.3390/plants15101458

**Published:** 2026-05-10

**Authors:** Songze Wu, Minghao Zhang, Ruicheng Hu, Di Niu, Boyu Meng, Haikun Yang, Yuling Chen, Yonghai Fan, Kun Lu

**Affiliations:** 1 Integrative Science Center of Germplasm Creation in Western China (Chongqing) Science City, College of Agronomy and Biotechnology, Southwest University, Chongqing 400715, China; 17781201791@163.com (S.W.); 18311870271@163.com (M.Z.); 15681922709@163.com (R.H.); niudi0321@163.com (D.N.); beyul1340516624@email.swu.edu.cn (B.M.); haikun_2000@163.com (H.Y.); yulingchen1823@163.com (Y.C.); 2 Engineering Research Center of South Upland Agriculture, Ministry of Education, Chongqing 400715, China

**Keywords:** trithorax-related, duplication, expression pattern, *ATXR6*, rapeseed

## Abstract

Histone methyltransferases of the *Trithorax-related* (*ATXR*) family act as critical epigenetic regulators in plants. However, systematic characterization of this gene family remains limited in the economically important oilseed crop *Brassica napus*. In this study, we performed a pan-genomic analysis of the *BnaATXR* family genes using 11 genetically diverse rapeseed accessions and identified a total of 185 *BnaATXR* family members, among which *BnaATXR5* was categorized as a dispensable gene. Pan-genomic and phylogenetic analyses grouped these genes into five distinct subfamilies and uncovered strong sequence conservation and pervasive purifying selection across the family. Whole-genome duplication (WGD) was identified as the major evolutionary force driving *BnaATXR* genes expansion. *Cis*-acting regulatory element analysis further revealed significant enrichment of stress- and phytohormone-responsive motifs in the promoter regions of *BnaATXR* genes. *BnaATXR* members exhibited divergent tissue expression profiles: subfamilies B and C displayed constitutive and broad expression across multiple tissues, whereas subfamilies A and E exhibited pronounced tissue-specific expression, with preferential enrichment in reproductive organs. Notably, CRISPR/Cas9-mediated knockout of *BnaATXR6* led to delayed flowering time, shortened siliques, and decreased seed size, thereby demonstrating a key functional role of this gene in the modulation of yield-associated agronomic traits. Collectively, our findings present a genome-wide systematic characterization of the *ATXR* gene family and highlight their critical functional relevance to agronomically important traits in rapeseed.

## 1. Introduction

Epigenetic regulation represents the fundamental mechanism governing gene expression and genomic stability in eukaryotes. Post-translational modifications of histones, such as histone lysine methylation, play an essential role in this biological process [1,2,3]. Epigenetic regulatory mechanisms are critical for plants to adapt to environmental challenges and maintain normal growth and development. Histone methylation is a key epigenetic modification in plants that modulates chromatin status and gene expression, thereby regulating diverse biological processes, including growth, development, and responses to external stimuli [4,5]. The SET-domain genes (SDGs) are associated with epigenetic regulation in plants and were the first identified for their roles in gene silencing and activation in *Drosophila melanogaster* [4,5]. The SET-domain proteins regulate the gene transcriptional activities by altering nucleosome structure via methyl group addition to lysine residues of histone H3 and H4 [5,6]. In plants, the SET-domain family is classified into seven major classes [4,6]: Class I: *E*(*Z*) homologs, which primarily catalyze histone H3 lysine 27 (H3K27) methylation and act in Polycomb repressive complex 2 (PRC2) to regulate plant development. Class II: *ASH1* homologs, which are responsible for histone H3 lysine 36 (H3K36) methylation and function in flowering time control and transcriptional regulation. Class III: *trithorax* (*Trx*) homologs and related proteins, which mainly mediate histone H3 lysine 4 (H3K4) methylation and serve as positive regulators of gene transcription. Class IV: proteins containing both a C-terminal SET domain and a plant homeodomain (PHD) finger. The PHD finger is a conserved zinc-finger domain widely found in chromatin-associated proteins and is capable of recognizing methylated histone marks. Class IV proteins (exemplified by ATXR5 and ATXR6) contribute to cell cycle regulation, DNA replication, and histone methylation via interactions with proliferating cell nuclear antigen (PCNA) and possess histone lysine methyltransferase activity. Class V: *Su*(*var*) homologs and relatives, which catalyze histone H3 lysine 9 (H3K9) methylation and mediate heterochromatin formation and transcriptional silencing. Class VI: proteins with truncated or interrupted SET domains, which may retain methyltransferase activity toward specific histone lysine residues. Class VII: non-histone substrate methyltransferases, represented by Rubisco large and small subunit methyltransferases (RBCMTs) that modify non-histone proteins.

The *ATXR* (*Trithorax-related*) family belongs to the plant SET domain family, with distinct members falling into Class I, Class III, and Class IV, and serves as a key regulator of epigenetic modifications in plants [1,6]. Plant *ATXR* genes originated in early land plants, and their homologs have been identified in bryophytes, including *Funaria hygrometrica* [7]. Among them, ATXR3 and ATXR7 belong to Class III Trithorax proteins and possess H3K4 methyltransferase activity, ATXR5 and ATXR6 belong to Class I E(Z) homologs and specifically catalyze H3K27me1 modification, and ATXR1 and ATXR2 proteins are grouped into Class IV; to date, few systematic studies have clearly elucidated the classification and functional characterization of ATXR4 [4,6]. In *Arabidopsis thaliana*, ATXR2 protein functions as a bidirectional regulator in cytokinin and auxin signaling cascades by mediating histone methylation modifications, including H3K36me3 [8]. It interacts with the cytokinin response factor RESPONSE REGULATOR 1 (ARR1) to activate type A *ARR* genes (e.g., *ARR5* and *ARR7*), while also repressing cytokinin signaling and restricting *WUSCHEL* (*WUS*) activation, thereby negatively regulating callus-to-shoot fate transition. Meanwhile, the ortholog of *ATXR2* in rice (*Oryza sativa*) also exhibits conserved functions analogous to its counterpart in *A. thaliana* [8]. Additionally, the ATXR2 protein promotes cell dedifferentiation and callus formation in *A. thaliana*. It cooperates with auxin response factors AUXIN RESPONSE FACTOR 7 (ARF7) and ARF19 to activate the expression of *LATERAL ORGAN BOUNDARIES-DOMAIN 16* (*LBD16*) and *LBD29* [9], two core transcription factors that mediate callus formation [10]. ATXR7 catalyzes H3K4me modification to activate the transcription of the flowering repressor *FLOWERING LOCUS C* (*FLC*) [11], and ATXR3 mediates genome-wide H3K4me3 modification [12,13]. The two proteins coordinately participate in the transcriptional activation and post-vernalization reactivation of *FLC* in *A. thaliana* [14,15].

As a conserved small subgroup within the SET domain gene family, the *ATXR* family has been identified and classified in multiple plant species, including *A. thaliana* [16], grape (*Vitis vinifera*) [17], maize (*Zea mays*) [18], rice [19], tomato (*Solanum lycopersicum*) [20], sweet orange (*Citrus sinensis*) [21], common wheat (*Triticum aestivum*) [22], and rapeseed [23]. In *Brassica* species, the *ATXR* family genes have been identified and are potentially involved in key agronomic traits, such as flowering through histone methylation in *Brassica rapa* [24]. However, systematic characterization of the *ATXR* family, especially its pan-genomic features and agronomic trait regulation, remains limited in rapeseed. Here, we identified *BnaATXR* genes from the rapeseed pan-genome across 11 accessions. To better elucidate the *BnaATXR* functions, we systematically analyzed their sequence characteristics, gene structure, promoter *cis*-acting elements, and tissue-specific expression patterns. Furthermore, we explored the functional characterization of *ATXR6* in rapeseed. Our study provides a comprehensive characterization of *ATXR* family genes and lays a foundation for dissecting their roles in rapeseed.

## 2. Results

### 2.1. Identification of BnaATXRs in Rapeseed Pan-Genome

**Figure 1:**
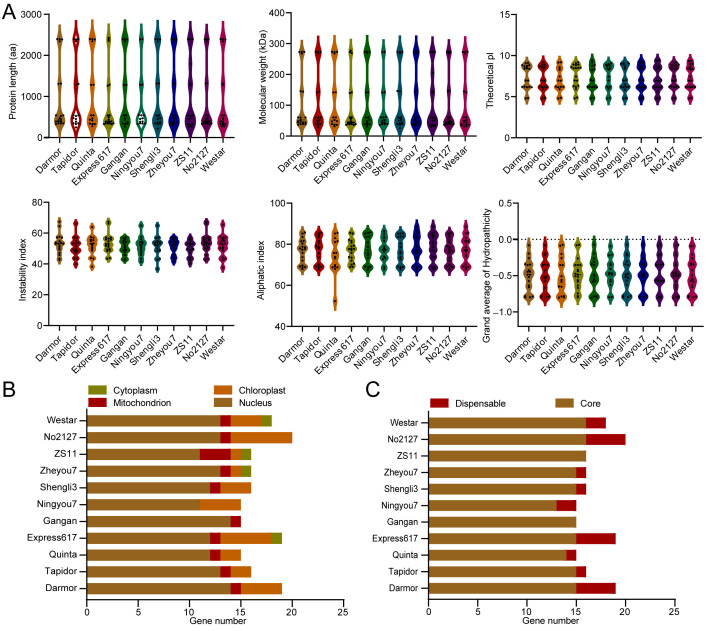
Characterization and classification of *BnaATXR* genes into core and dispensable genes across 11 rapeseed genomes. (**A**) Characterization of BnaATXR proteins, including protein length, molecular weight, theoretical isoelectric point (pI), instability index values and average hydropathicity (GRAVY) values. (**B**) Subcellular localization distribution of BnaATXR proteins. (**C**) Proportions of core and dispensable *BnaATXRs* across the 11 rapeseed genomes.

In total, we identified 185 BnaATXR protein members across 11 high-quality rapeseed genomes using seven ATXR protein sequences in *A. thaliana* as queries, and each rapeseed genome contained approximately 15–20 *BnaATXR*-encoding genes (Table 1 and Table S1). The morphological characteristics of rapeseed accessions for pan-genome have been well described in previous studies [25]. Among them, the spring-ecotype No2127 exhibited the highest number of *BnaATXR* genes (20 genes), while the winter-ecotype Quinta, semiwinter-ecotype Gangan/Ningyou7 had the lowest with 15 *BnaATXR* genes (Table 1). The lengths of the BnaATXR protein sequences in rapeseed ranged from 222 (Shengli3, BnaC07.ATXR6) amino acids (aa) to 2425 aa (Express617, BnaC01.ATXR3), with an average of 947 aa (Figure 1A, Table S1). The molecular weights (MWs) of ATXR proteins ranged from 25.37 (Shengli3, BnaC07.ATXR6) to 277.31 kDa (Express617, BnaC01.ATXR3), averaging 107.01 kDa, while the isoelectric points (pIs) varied from 4.78 (BnaA05.ATXR2 in ZS11, No2127, Quinta, Ningyou7 and Shengli3) to 9.42 (BnaA02.ATXR6 in Westar, ZS11 and Gangan), with an average of 7.30 (Figure 1A, Table S1). In total, 122 of the 185 BnaATXR proteins (65.95%) had an instability index value greater than 50, indicating that most BnaATXRs are unstable proteins. All BnaATXR proteins showed values less than 0 for the grand average of hydropathicity, suggesting their hydrophilic nature. Moreover, no signal peptide was found in the BnaATXR proteins, only one protein (BnaC01.ATXR3 in Express617) contained transmembrane helices, and most BnaATXR proteins were predicted to localize in the nucleus (Figure 1B, Table S1).

We compared the copy-number variations (CNVs) in the *BnaATXR* family genes between rapeseed accessions. *BnaATXR5* was absent in semiwinter-ecotype Gangan and ZS11, but four copies were present in winter-ecotype Darmor/Express617 and spring-ecotype No2127, and two copies in semiwinter-ecotype Ningyou7 and spring-ecotype Westar (Table 1), indicating CNVs between rapeseed accessions. Furthermore, we identified the core gene and dispensable gene families in the rapeseed pan-genome, while the 20 *BnaATXR5* genes were assigned to two dispensable gene families, whereas the remaining *BnaATXRs* were grouped into nine core gene families (Figure 1C). Rapeseed is an allopolyploid (AACC genome) derived from the *Brassica rapa* (AA) and *Brassica oleracea* (CC). We identified the *ATXR* genes from its diploid parents. The results show that the four *B. rapa* genomes contained 8–10 *ATXR* genes, and the four *B. oleracea* genomes contained 10–11 *ATXR* genes (Tables S2 and S3). The number of *ATXR* members from the *B. napus* subgenomes was approximately consistent with that of its diploid parents, suggesting that *BnaATXRs* are evenly distributed across both subgenomes in *B. napus.*

### 2.2. Phylogenetic Classification of ATXRs in Brassica

**Figure 2:**
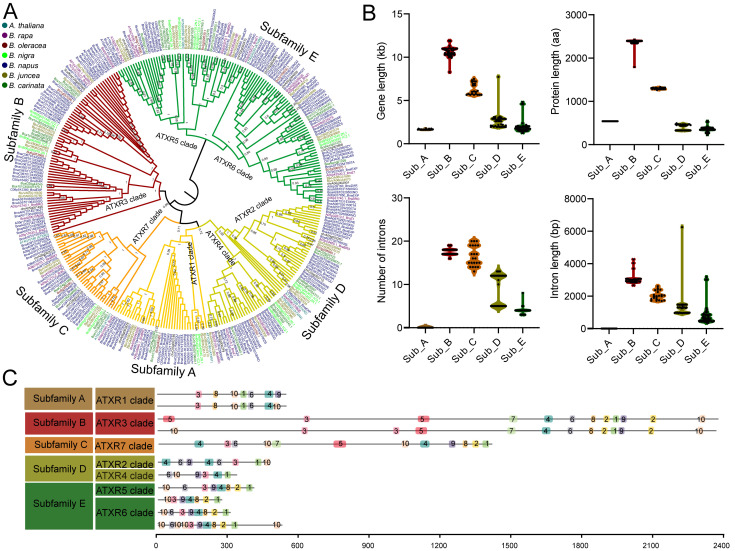
Phylogenetic classification, gene structure features, and conserved motif analysis across ATXR subfamilies. (**A**) Phylogenetic tree constructed using protein sequences of ATXR members from 25 *Brassica* genomes and 7 ATXR members from *A. thaliana*. The tree was built in MEGA11 using the NJ method with p-distance model and 1000 bootstrap replicates, and branches with bootstrap values ≥ 50% were visualized in FigTree. (**B**) Gene length, protein length and intron length distribution across *BnaATXR* subfamilies. (**C**) Distribution of 10 conserved motifs identified by MEME across BnaATXR proteins.

To explore the evolutionary relationship of *ATXR* genes in U’s triangle *Brassica* species (which comprises three basic diploid plant species—*B. rapa*, *B. oleracea*, and *Brassica nigra*—and three allopolyploid species—*B. napus*, *Brassica juncea*, and *Brassica carinata*), we additionally identified *ATXR* genes in two *B. nigra* (BB) genomes, two *B. juncea* (AABB) genomes and two *B. carinata* (BBCC) genomes. In total, 354 *ATXR* genes were identified across the 25 U’s triangle *Brassica* genomes. Using ATXR protein sequences from *A. thaliana* and six *Brassica* species, a phylogenetic tree was constructed via the neighbor-joining (NJ) method. The results show that these ATXR proteins could be grouped into five distinct subfamilies based on bootstrap support values: subfamily A (ATXR1 clade), subfamily B (ATXR3 clade), subfamily C (ATXR7 clade), subfamily D (ATXR2/ATXR4 clades), and subfamily E (ATXR5/ATXR6 clades) (Figure 2A). Subfamily E represented the largest clade, containing 125 ATXR protein members from *Brassica* species, followed by subfamily B (77 members) and subfamily D (73 members) (Figure 2A, Table 1). Subfamily A and subfamily C were the smallest clades, harboring 39 and 40 *Brassica* ATXR protein members, respectively. ATXR members were present in all subfamilies across all tested species. Compared with the three diploid progenitor species (*B. rapa*, *B. nigra*, *B. oleracea*), *ATXR* genes exhibited no obvious gene family expansion in the three allopolyploid species (*B. juncea*, *B. carinata*, and *B. napus*), although extensive CNVs were observed between individual accessions, such as those of *ATXR5* in *B. napus* and *B. oleracea* (Tables S1–S6). These findings indicate that *ATXR* gene duplication events occurred prior to allopolyploidization in *Brassica*. Furthermore, no lineage-specific subfamily expansion was detected among the six investigated *Brassica* species.

To further explore the evolutionary features of rapeseed *BnaATXR* genes, we analyzed their gene structures and conserved protein motifs. The coefficient of variation (CV) of the gene length was below 10% in subfamilies A (3.14%, average length 1656 bp) and B (5.47%, average length 10,622 bp), followed by subfamily C (10.75%, average length 6328 bp), while subfamilies D (36.07%, average length 2637 bp) and E (38.75%, average length 1941 bp) exhibited extensive variations in gene length, suggesting a weak subfamily-specific trend in gene length (Figure 2B). The protein sequence lengths of subfamilies A, B, and C were also conserved, with CVs ranging from 0.08 to 3.98%, while subfamilies D and E had more variable protein lengths, with CVs ranging from 16.22 to 17.02%. Interestingly, no introns were found in subfamily A, and more than 10 introns were found in subfamilies B and C, which was similar to the variation in intron length. Moreover, the intron variations were also similar to those of gene length and protein length variations, suggesting that the *BnaATXR* gene length variation within subfamilies primarily originates from differences in introns in rapeseed.

Motif analysis revealed that motifs 3, 1, and 4 were highly conserved across all subfamilies, and motif 1 corresponded to the canonical SET domain (Figure 2C). Motif 2 was conserved in subfamilies B, C, and E, while motifs 5 and 7 were unique to subfamilies B and C, respectively. Motif 6 was present in most subfamilies, except for several ATXR6 clade members within subfamily D. Similarly, motif 8 was absent from subfamily B, and motifs 9 and 10 were not detected in a subset of members of subfamily A. These results illustrated that the BnaATXR protein sequences remain relatively conserved within each subfamily, whereas distinct differences in intron structure and motif composition likely drive the functional diversification of *BnaATXR* genes in rapeseed.

### 2.3. The Expansion Mechanism of BnaATXRs

**Figure 3:**
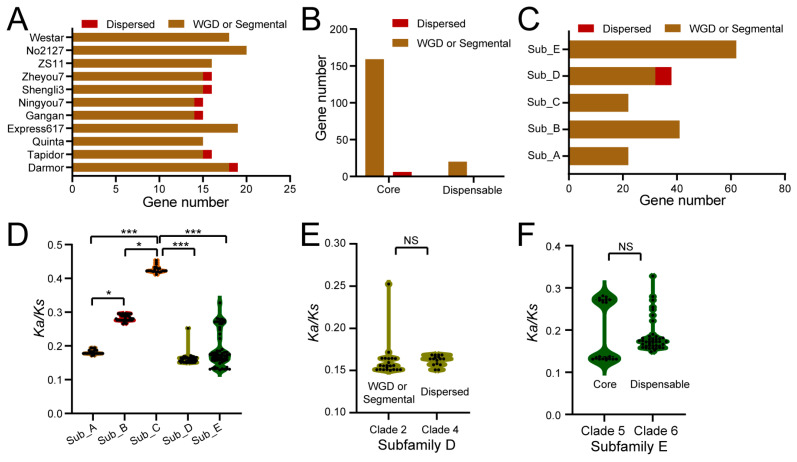
Duplication event classification and selection pressures among *BnaATXR* genes in rapeseed. (**A**) Distribution of different types of gene duplication events between *BnaATXR* genes in rapeseed genomes. (**B**) Number of core and dispensable *BnaATXR* genes associated with each duplication type. (**C**) Number of genes assigned to each duplication type across subfamilies. (**D**) *Ka*/*Ks* ratios of *BnaATXR* genes in subfamilies. * *p* < 0.05, *** *p* < 0.001, Student’s *t*-test. (**E**) *Ka*/*Ks* ratios of *BnaATXR* genes in subfamily D. NS, *p* > 0.05, Student’s *t*-test. (**F**) *Ka*/*Ks* ratios of *BnaATXR* genes in subfamily E. NS, *p* > 0.05, Student’s *t*-test.

To explore the expansion mechanisms of *BnaATXRs* in rapeseed, we examined gene duplication patterns across the rapeseed pan-genome. The results show that the 185 *BnaATXRs* were assigned to two duplication types, 179 genes arose from WGD/segmental duplication, while the remaining six genes underwent dispersed duplication (Figure 3A–C). No proximal or tandem duplication or singletons was found in *BnaATXRs*, suggesting that WGD/segmental duplication was the predominant expansion mechanism for *BnaATXRs* in rapeseed genome. The occurrence of dispersed duplication event was detected in the core *BnaATXR4* gene in the Zheyou7, Shengli3, Ningyou7, Gangan, Tapidor and Darmor genomes, where each of these genomes harbors only one *BnaATXR4* copy (Figure 3A–C, Table 1). By comparison, *BnaATXR4*-clade in other genomes (carrying two *BnaATXR4* copies) belonged to WGD/segmental duplication. Moreover, the expansion of dispensable *BnaATXR5*-clade was also driven primarily by WGD/segmental duplication in rapeseed (Figure 3A–C).

### 2.4. Selection Pressures on BnaATXRs Orthologs

Selection pressure is the evolutionary force shaping genetic variation, driving adaptation and maintaining functional constraints among species. Here, we assessed the ratio of nonsynonymous (*Ka*) to synonymous (*Ks*) substitution between *A. thaliana* and each rapeseed genomes. A total of 185 *BnaATXR* gene pairs were identified. The results of *Ka*/*Ks* ratios of orthologous genes illustrated that all *BnaATXR* genes across the 11 rapeseed genomes experienced purifying selection (*Ka*/*Ks* < 1) (Figure 3D), suggesting that predominant purifying selection has maintained core methyltransferase functions during evolution. Notably, the subfamily C members (*BnaATXR*7 copies) exhibited the highest average *Ka*/*Ks* ratio (0.43) among all subfamilies, which was significantly higher than those in other subfamilies (Figure 3D), suggesting relatively weaker selective constraints on *BnaATXR*7 copies. Moreover, the CV of *Ka*/*Ks* was below 4% in subfamilies A (3.68%) and B (3.34%), followed by subfamily C (2.36%), while subfamilies D (10.27%) and E (27.33%) exhibited extensive variation in *Ka*/*Ks* ratios, indicating a weak subfamily-specific trend in selection pressures. In addition, we also compared the *Ka*/*Ks* values between WGD/segmental and dispersed duplication genes in subfamily D and between core and dispensable genes in subfamily E, but no significant difference was detected between these groups, whether for duplication events or for core and dispensable genes (Figure 3E,F).

### 2.5. Analysis of BnaATXRs Promoter Cis-Acting Elements

**Figure 4:**
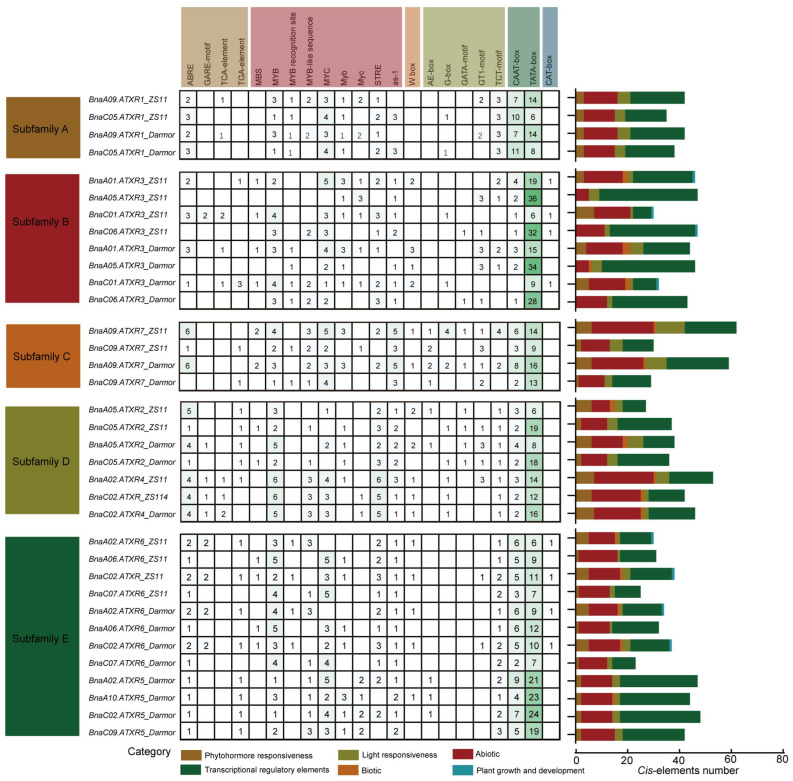
Summary of *cis*-acting elements in the promoter regions of *BnaATXR* genes in the ZS11 and Darmor genomes.

*Cis*-acting elements play critical roles in regulating gene transcription and expression. Here, we analyzed the promoter regions of *BnaATXR* genes in ZS11 and Darmor genomes. The promoter regions of *BnaATXR* genes (2000 bp upstream of the ATG start codon) had a number of *cis*-acting elements associated with various biological processes. After excluding the components with general transcriptional regulatory elements—such as TATA-box, CAAT-box and AT~TATA; non-Brassicaceae-specific elements; and elements with unknown functions—the remaining *cis*-acting elements were mainly divided into five categories (Figure 4): abiotic stress, biotic stress, light response, phytohormone response, and growth and development elements. Members of different *BnaATXR* subfamilies exhibited significant differences in the number and composition of cis-acting elements, while the distribution patterns of these elements were highly conserved among members within the same subfamily (Figure 4). For example, *BnaATXR* genes in subfamilies C and D contained a variety of *cis*-acting elements related to light responsiveness, while the CAT-box (involved in plant growth and development) was only found in subfamilies B and E. Moreover, orthologous *BnaATXR* genes from the ZS11 and Darmor genomes exhibited a high degree of overall conservation in the abundance and distribution of their promoter cis-regulatory elements, suggesting that the expression regulatory mechanisms of this family are evolutionarily conserved among diverse rapeseed accessions. Among the functionally relevant cis-elements, abiotic stress-responsive elements were the most abundant in the promoter regions of *BnaATXR* genes. In particular, a large set of broadly acting abiotic stress-responsive elements, including MBS, MYB, and MYC (which confer drought responsiveness), were highly enriched in the promoters of most *BnaATXR* genes, suggesting that *BnaATXR* genes may be widely involved in the response of rapeseed to drought and other abiotic stresses. Interestingly, the promoter regions of all *BnaATXR* genes contained at least one light response element, and the majority carried phytohormone response elements (Figure 4), particularly the ABRE element (responsible for abscisic acid responsiveness). These findings suggest that *BnaATXR* genes play an important role in integrating light and hormonal signaling pathways in rapeseed.

### 2.6. Expression Patterns of BnaATXRs in Rapeseed

**Figure 5:**
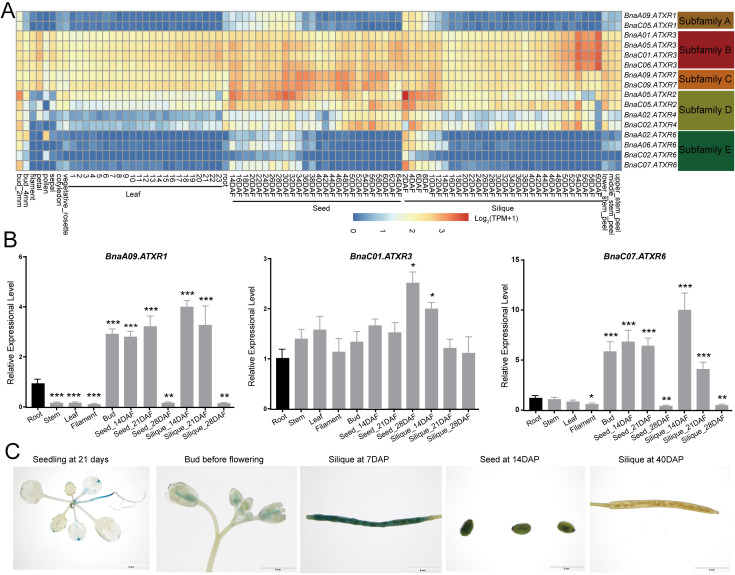
Expression patterns of *BnaATXR* genes in rapeseed. (**A**) Expression patterns of *BnaATXRs* in different organs and development stages from public transcriptome data. The gene expression level is shown on a graded color scale according to Log_2_ (TPM + 1) values. (**B**) Expression patterns of *BnaATXRs* in different organs and development stages verified by qRT-PCR. * *p* < 0.05, ** *p* < 0.01, *** *p* < 0.001, with three technical replicates per sample, Student’s *t*-test. (**C**) Histochemical analysis of GUS activity in *A. thaliana* plants expressing *ATXR6pro:GUS*. DAP: days after pollination.

We collected transcriptome datasets from the *Brassica napus* multi-omics information resource (BnIR) for *BnaATXR* genes (ZS11) across 91 tissues, as well as under abiotic stress and exogenous stimuli. *BnaATXR* genes from different subfamilies exhibited distinct expression patterns, while members within the same subfamily showed similar expression patterns (Figure 5A). The subfamilies B and C members exhibited broad expression patterns, and all of them were highly expressed in all examined tissues. Similarly, *BnaATXR* genes in subfamily D also exhibited high expression levels in most tissues, especially the two *BnaATXR4* copies. In contrast, members in subfamilies A and E exhibited tissue-specific expression patterns, and these *BnaATXRs* were preferentially expressed in bud, and the early stages of seed and silique development (Figure 5A).

To examine the reliability of the transcriptome data, we performed qRT-PCR for three genes (*BnaA09.ATXR1*, *BnaC01.ATXR3*, *BnaC07.ATXR6*) in 11 tissues (see Materials and Methods). The qRT-PCR verification results showed that *BnaA09.ATXR1* and *BnaC07.ATXR6* both preferred to be expressed in buds, and the early stages of seed and silique development, while *BnaC01.ATXR3* exhibited no significant differences in most detected tissues (Figure 5B), which was consistent with the expression pattern obtained from the transcriptome data (Figure 5A). Moreover, we also detected the expression profile of *BnaC07.ATXR6* by transforming *ATXR6pro:GUS* into *A*. *thaliana*. GUS staining of homozygous transgenic T_2_ plants showed that *BnaC07.ATXR6* was highly expressed during seed and silique development (Figure 5C), further confirming the reliability of the public transcriptome data used in this study.

### 2.7. BnaATXRs Respond to Abiotic Stress and Exogenous Phytohormones Treatments in Rapeseed

**Figure 6:**
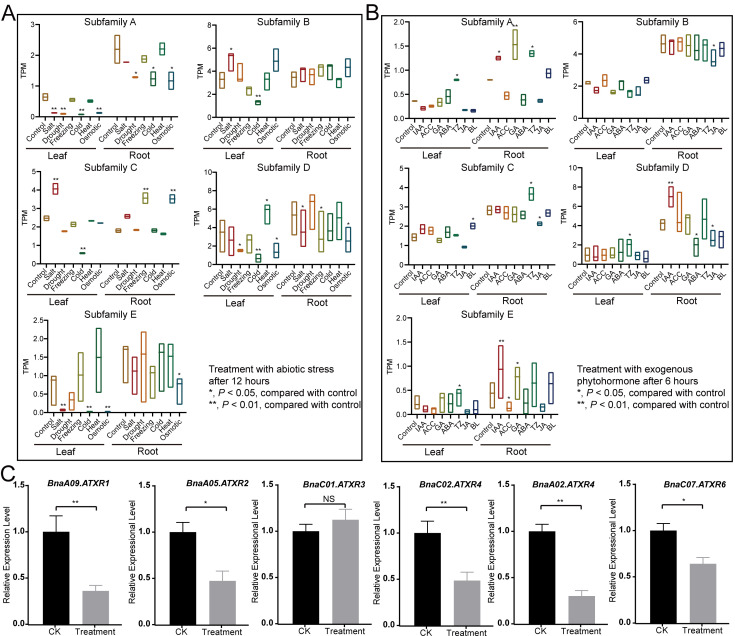
Expression patterns of *BnaATXR* in rapeseed under abiotic stress and exogenous phytohormones treatments. (**A**) Expression profiles of *BnaATXRs* in response to abiotic and biotic stress. Control: control group, Salt: salt treatment, Drought: drought treatment, Freezing: freezing treatment, Cold: cold treatment, Heat: heat treatment, Osmotic: osmotic treatment. (**B**) Expression profiles of *BnaATXRs* in response to phytohormones. IAA: indole-3-acetic acid, ACC: 1-Aminocyclopropane-1-carboxylic acid, GA: gibberellic acid, ABA: abscisic acid, TZ: trans-zeatin, JA: jasmonic acid, BL: brassinolide. (**C**) Expression patterns of six *BnaATXR* genes exposed to corresponding drought treatments in rapeseed (ZS11). * *p* < 0.05, ** *p* < 0.01, NS: no significance, with three technical replicates per sample, Student’s *t*-test.

To explore the response to abiotic stress and exogenous phytohormones, we also analyzed the expression patterns of *BnaATXR* genes under abiotic stress and exogenous phytohormone treatments. Most *BnaATXRs* were repressed when subjected to abiotic stress treatments (Figure 6A), including members of subfamilies A, D, and E in leaf and root. In contrast, *BnaATXR* genes from subfamilies B and C were induced in leaves under salt stress, and in roots under freezing and osmotic stresses. Genes from subfamily D were repressed in leaves after the drought treatment. However, some *BnaATXR* genes showed altered expression in response to certain phytohormones (Figure 6B): for example, *BnaATXR* genes from subfamily A responded to IAA and TZ in roots; those from subfamilies A and D responded to TZ in leaves; and those from subfamily E responded to IAA, ACC and GA in roots. Overall, *BnaATXR* genes exhibited stronger responses to abiotic stresses, which was consistent with the distribution of *cis*-acting elements in their promoter regions. Moreover, qRT-PCR verification confirmed that the expression levels of six genes (*BnaA09.ATXR1*, *BnaA05.ATXR2*, *BnaC01.ATXR3*, *BnaC02.ATXR4*, *BnaA02.ATXR4*, and *BnaC07.ATXR6*) were downregulated under the drought treatment (Figure 6C).

### 2.8. Functional Characterization of BnaATXR6 in Rapeseed

**Figure 7:**
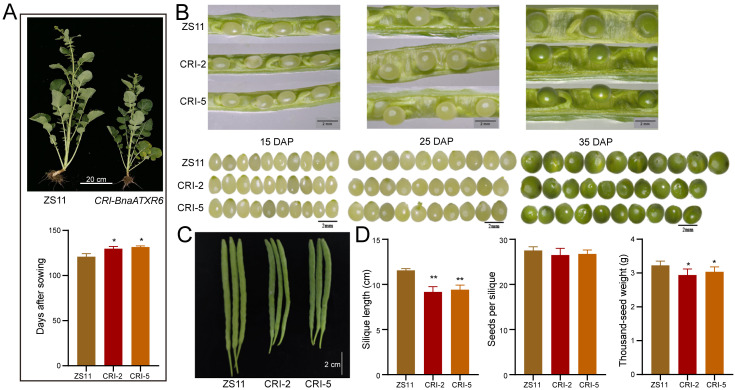
Phenotypes of ZS11 and *CRI-BnaATXR6* transgenic plants. (**A**) Comparison of flowering between ZS11 and *CRI-BnaATXR6* transgenic plants. * *p* < 0.05, Student’s *t*-test. (**B**) Seed development of ZS11 and *CRI-BnaATXR6* transgenic plants in different stages, scale bar: 2 mm. (**C**) Silique phenotype of ZS11 and *CRI-BnaATXR6* transgenic plants during mature stage. (**D**) Statistical analysis of silique length, seeds per silique, and thousand-seed weight between ZS11 and *CRI-BnaATXR6* transgenic plants, * *p* < 0.05, ** *p* < 0.01, *n* = 9, Student’s *t*-test.

Due to the tissue-specific expression patterns of subfamilies A (*ATXR1* copies) and E (*ATXR6* copies), we explored the functional characterization of *BnaATXR6* in rapeseed. Using the CRISPR/Cas9 gene editing method, we successfully obtained 13 T_0_ generation transgenic lines. We examined the target gene efficiency via HI-TOM sequencing and identified six edited lines carrying single nucleotide polymorphisms (SNPs) or insertion-deletion (Indel) variations (editing efficiency: 46.15%). Finally, we selected line 2 (CRI-2, termination encoding) and line 5 (CRI-5, displacement encoding) for phenotypic examination based on their SNP or Indel variation annotations (Figure S1). Compared with wild-type ZS11, the T_2_ generation of *BnaATXR6*-edited plants exhibited delayed flowering (Figure 7A), smaller seed size (Figure 7B), and reduced silique length (Figure 7C). Notably, the number of seeds per silique remained unchanged between the ZS11 (control) and the transgenic lines (Figure 7D), suggesting that *BnaATXR6* primarily acts as a positive regulator of silique and seed morphology in rapeseed.

## 3. Discussion

In this study, we conducted a comprehensive characterization of the *BnaATXR* family genes in the rapeseed pan-genome. Our analyses provided valuable insights into the evolutionary dynamics, structural features, and putative functional roles of *BnaATXR* genes in rapeseed.

### 3.1. Evolutionary Conservation and Divergence of the BnaATXR Family in Rapeseed

We identified 185 *BnaATXR* members across 11 accessions (15–20 genes per genome), confirming the expansion and diversification of this gene family in the allopolyploid *B. napus* (Table 1). A previous study identified 20 *BnaATXR* genes from Darmor genome (V5) [22], while 19 *BnaATXR* genes were identified from the Darmor genome (V10) in this study. The discrepancy arises because we identified only one *BnaATXR4* copy, whereas two *BnaATXR4* copies were detected in the previous study, suggesting that the different versions of the Darmor genome assembly likely caused this difference in *BnaATXR* gene identification. Moreover, variations in assembly quality may also lead to the absence of certain genes [25]. For example, copy number variations (CNVs) of *BnaATXR5* (0–4 copies between ecotypes) suggest a potential link between dispensable gene-mediated genomic plasticity, which might be consistent with the role of epigenetic regulators in plant adaptation [2,3,23] or with differences in assembly quality. In addition, phenotypic differences between the rapeseed accessions used in this pan-genome study [25] suggest a potential role of CNVs in *BnaATXR* as epigenetic regulators in rapeseed, though further experiments are required to explore this relationship.

Gene average length is a fundamental genomic feature that reflects evolutionary divergence, structural complexity, and functional differentiation within a gene family [26]. Variation in gene length across subfamilies directly mirrors the evolutionary divergence and adaptive differentiation of gene family members [27]. In this study, the distinct patterns of gene length variation between *BnaATXR* subfamilies were mainly determined by non-coding regions rather than coding sequences. Further structural comparison confirmed that intron length polymorphism, rather than exon length variation, was the primary contributor to gene length divergence in *BnaATXR* genes. Subfamilies with longer average gene lengths harbored abundant and longer introns, while subfamilies D and E exhibited highly variable intron sizes, resulting in large fluctuations in total gene length. By contrast, the exon sequences encoding conserved functional domains remained relatively conserved in both length and composition across all subfamilies. Conserved exon architecture ensures the maintenance of core methyltransferase activity, whereas flexible intron variation serves as a major driving force for gene family expansion, subfamily functional differentiation, and transcriptional regulatory diversity. Intron-level variation also provides a genetic basis for alternative splicing and stress-responsive expression adjustment, which may help rapeseed *BnaATXR* members cope with complex growth and environmental conditions.

Phylogenetic analysis clustered *Brassica* ATXR proteins into five subfamilies (A–E), each showing a strong correspondence to *A*. *thaliana* homologs, highlighting their evolutionary conservation. The classification of *Brassica* ATXR proteins also corresponded to the classification of *Brassica* SDG proteins [23], in which ATXR5/6 members clustered together, ATXR1/2/4 members showed a close relationship, and ATXR3 and ATXR7 members formed distinct individual clusters. Notably, while gene numbers did not show marked expansion in allopolyploids compared with their diploid progenitors (Tables S1–S6), and the *BnaATXR* family genes expanded largely through WGD and segmental duplication processes. This pattern aligned with the genomic history of *Brassica* species, where allopolyploidization drives gene family amplification, followed by homoeologous exchanges that can reshape gene dosage and foster functional diversification [28,29,30,31]. Purifying selection acting on all *BnaATXR* orthologs indicated strong evolutionary constraints to maintain core methyltransferase functions, as previously highlighted for *ATXR* genes in *A. thaliana* [1]. However, the relatively higher average *Ka*/*Ks* ratio in subfamily C (*BnaATXR7* copies) pointed to potentially relaxed selective constraints, and these genes exhibited broad tissue-specific expression patterns in all examined tissues. These results highlighted that *BnaATXR7* family members might have undergone moderate functional divergence in a sustained manner, allowing for potentially evolving roles in epigenetic regulation or functional adaptation in rapeseed.

### 3.2. Expression Patterns and Putative Roles in Stress Response and Development

Promoter *cis*-acting element analysis and expression profiling collectively indicated that *BnaATXR* genes are potential components of signaling networks regulating plant development and stress responses. Our analysis revealed an enrichment of abiotic stress-responsive elements in *BnaATXR* promoters. Consistent with this, several *BnaATXR* genes were significantly downregulated under drought stress, suggesting their direct role in drought response pathways. This aligned with the established role of histone methylation in mediating transcriptional reprogramming under environmental stresses [2,3]. Light-responsive elements were found in all *BnaATXR* genes, and subfamilies B and C showed broad and high expression in various tissues. The broad expression pattern of *BnaATXR* homologs might be consistent with their known function in modulating the flowering repressor *FLC* via H3K4me in *A. thaliana* [11,14]. In contrast, the tissue-specific expression of subfamilies A (*BnaATXR1* copies) and E (*BnaATXR5*/6 copies) in buds and early reproductive tissues, coupled with their lower protein instability indices, suggests specialized functions in reproductive development [7,15], which will be further investigated. Unstable proteins were readily degraded via the ubiquitin-proteasome system, thereby enabling dynamic regulation of histone methylation levels in response to abiotic stress and phytohormone signals [32]. We found that 65.95% BnaATXR proteins were unstable proteins, which provided a mechanistic basis for rapid epigenetic regulation in rapeseed. However, these predictions from *BnaATXR* promoters need further validation.

### 3.3. Functional Validation Implicates BnaATXR6 in Regulating Yield-Related Traits

Due to the tissue-specific expression patterns of *BnaATXR1* and *BnaATXR6* copies in buds and early reproductive tissues, we explored the functional characterization of *BnaATXR* genes using *BnaATXR6* as the representative gene. Our functional characterization of *BnaATXR6* provided preliminary evidence for its role in regulating agronomically important traits in rapeseed. The CRISPR/Cas9-generated *CRI-BnaATXR6* mutants exhibited delayed flowering, shorter siliques, and reduced seed size, establishing *BnaATXR6* as a potential positive regulator of yield-related traits in rapeseed. The delayed flowering phenotype paralleled the function of its *A. thaliana* homologs *ATXR7* and *ATXR3* in repressing flowering through *FLC* activation [11,14], indicating a conserved function in flowering time regulation. Notably, although the CRISPR/Cas9 editing sites were located in the coding region rather than the promoter sequence of *BnaATXR6*, we observed significantly altered transcript abundance of *BnaATXR6* in edited lines (Figure S1). This expression fluctuation independent of promoter mutation could be reasonably explained by multiple internal factors. For example, coding-region mutations may trigger nonsense-mediated mRNA decay or reduce transcript stability, thereby decreasing gene expression at the post-transcriptional level. Moreover, the amino acid mutations caused by Indel or SNP variations may disrupt protein structure and epigenetic modification activity, further inducing feedback regulation of gene transcription. As core epigenetic regulators, *ATXR* family members are involved in complex chromatin modification networks, and the loss-of-function of *BnaATXR6* may indirectly affect the expression of downstream or homologous genes through epigenetic feedback loops. Thus, the novel finding on silique and seed size regulation suggested a potential mechanism: *BnaATXR6*-mediated histone methylation (potentially H3K27me1, as reported in other species [7]) may influence pathways controlling cell expansion or hormonal signaling during seed and silique development. This parallels the interaction between ATXR2 and auxin response factors (ARF7/ARF19) to promote callus formation in *A. thaliana* [9], highlighting the broader roles of ATXR proteins in integrating epigenetic control with hormone signaling to regulate plant development.

## 4. Materials and Methods

### 4.1. Identification of BnaATXR Genes in Rapeseed Pan-Genome

Genomic, coding, and proteomic sequences of *A. thaliana* were obtained from the *A*. *thaliana* Information Resource (TAIR, http://www.arabidopsis.org, accessed on 4 January 2026); those for rapeseed accessions (including Darmor, Tapidor, Quinta, Express617, Gangan, Ningyou7, Shengli3, Zheyou7, ZS11, No2127 and Westar) were downloaded from the *Brassica napus* multi-omics information resource (BnIR, http://yanglab.hzau.edu.cn/BnIR, accessed on 6 January 2026); those for *B. rapa* accessions (Chiifu, Z1, BRO and ECD04), *B. nigra* accessions (CN115125 and NI100) and *B. juncea* accessions (AU213 and Tumida) were obtained from the *Brassica juncea* Information Resource (BjuIR, https://yanglab.hzau.edu.cn/BjuIR/, accessed on 4 February 2026); and those for *B. oleracea* accessions (TO1000, HDEM, Korso and OX-heart) and *B. carinata* accessions (10167 and zd-1) were downloaded from BnaOmics (https://bnaomics.ocri-genomics.net/, accessed on 4 February 2026) and the Brassicaceae Database (http://www.brassicadb.cn/#/, accessed on 4 February 2026). To identify *ATXR* genes in *Brassica* species, reciprocal Basic Local Alignment Search Tool Protein (BLASTP) (v2.2.30) [33,34] analysis was performed with seven ATXR protein sequences as the query sequences at a threshold E-value of 1 × 10^−5^ and a minimum alignment coverage of 50% [35]. Then, all candidate ATXR proteins were analyzed with Pfamscan (https://jobdispatcher.org/pfamscan, accessed on 10 February 2026) to identify the presence of the SET domain (PF00856.34).

### 4.2. Characteristic Analysis of BnaATXR Protein Sequences

The physicochemical properties of BnaATXR protein sequences were analyzed using the ExPASy ProtParam tool (https://web.expasy.org/protparam/, accessed on 12 February 2026) [36], including the amino acid length, molecular weight, isoelectric point, instability index, aliphatic index, and hydropathicity. The transmembrane transport peptides of proteins were predicted using the TMHMM-2.0 tool (https://services.healthtech.dtu.dk/services/TMHMM-2.0/, accessed on 12 February 2026) [37], and the signal peptides were conducted using SignalP5.0 (http://www.cbs.dtu.dk/services/SignalP/, accessed on 12 February 2026) [38], with default parameters. The subcellular localization predictions of proteins were performed with the online WoLF PSORT platform (https://wolfpsort.hgc.jp/, accessed on 12 February 2026) [39].

### 4.3. Phylogenetic and Evolutionary Analyses

Multiple-sequence alignment of ATXR protein sequences was conducted using Molecular Evolutionary Genetics Analysis (MUSCLE V5 [40], University of Michigan, Ann Arbor, MI, USA) with default parameters. Then, the phylogenetic tree was constructed by Molecular Evolutionary Genetics Analysis11 (MEGA11 [41], Tokyo Metropolitan University, Tokyo, Japan) with neighbor-joining (NJ) method, with the p-distance model, 1000 bootstrap replications and pairwise deletion option for missing data. The phylogenetic tree was visualized using FigTree (v1.4.1, http://tree.bio.ed.ac.uk/software/figtree/, accessed on 22 February 2026). Gene family clustering across the 11 rapeseed genomes was analyzed using Orthofinder (v2.3.1) [42], with default parameters to classify core (present in all 11 genomes), dispensable (present in 2–10 genomes), and private (present in one genome) *ATXR* genes.

### 4.4. Gene Structure, Motif, and Duplication Events Identifications

The Gene Structure Display Server (GSDS 2.0: https://gsds.gao-lab.org/, accessed on 23 February 2026) was used to analyze the exon/intron structures of the *ATXR* genes. Conserved motifs in rapeseed BnaATXR proteins were identified using the Multiple Expectation Maximization for Motif Elicitation program (MEME 4.12.0, https://meme-suite.org/meme/doc/download.html, accessed on 20 February 2026) with the following parameter settings: the minimal and maximal motif widths were set to 6 and 100 amino acids, and the maximum number of motifs was set to 10; the results were visualized with TBtools (v2.44.1). Gene duplication events of *ATXR* genes were identified using the Multiple Collinearity Scan toolkit (MCScanX v1) [43] with the following parameters: match_score: MATCH_SIZE: 5, gap_penalty: −1, overlap_window: 5, E_value: 1 × 10^−5^, max_gaps: 25. The duplication events were classified into whole-genome duplication (WGD), segmental, dispersed, tandem, and proximal duplications.

### 4.5. Selection Pressures Analysis

The CDS of *ATXR* orthologs from *A. thaliana* and rapeseed first aligned using MUSCLE and then grouped into 185 gene pairs. The KaKs_calculator 2.0 software [44] was used to calculate the synonymous mutation rate (*Ks*), non-synonymous mutation rate (*Ka*), and evolutionary constraint (*Ka*/*Ks*), where *Ka*/*Ks* > 1 indicates positive selection, *Ka*/*Ks* = 1 indicates neutral selection, and *Ka*/*Ks* < 1 indicates purifying selection.

### 4.6. Promoter Cis-Acting Elements and Expression Patterns Analysis

The 2000 bp upstream sequences (up to -ATG) of *ATXR* genes extracted with TBtools, then predicted of *cis*-acting elements using the online tool PlantCARE (https://bioinformatics.psb.ugent.be/webtools/plantcare/html/, accessed on 24 January 2026). The expression data for *BnaATXR* in rapeseed cultivar Zhongshuang11 (ZS11) were retrieved from the BnIR, and the expression data were corrected for batch effects with three repeats per tissue. There were 91 different tissues, including roots, stems, leaves and sepals, petals, pollen, siliques and seeds at different stages, as shown in Figure S2. Furthermore, the expression patterns of *ATXR* genes under phytohormone and abiotic treatments at the seedling leaves and roots were also analyzed. All gene expression levels were normalized by Log_2_(TPM + 1).

### 4.7. Vector Construction, Plant Transformation, GUS Histochemical Staining and Phenotypic Observation

The 2000-bp genomic sequence upstream of *BnaC07.ATXR6* was amplified to replace the Camv35S promoter in the *pCAMBIA1305.1* vector, generating *ATXR6pro:GUS*, and transgenic *A*. *thaliana* lines were obtained using Agrobacterium tumefaciens-mediated transformation with the strain GV3101. The GUS staining of transgenic *A*. *thaliana* lines was described in our previous study [45]. Construction of the double-target CRISPR/Cas9 rapeseed vector was performed according to the method previously described by Xing et al. [46], the *pYLCRISPR/Cas9* vector was used to construct editing vectors, and all primer sequences used for vector construction were listed in Table S7. Transgenic rapeseed (ZS11 background) lines were generated using *Agrobacterium tumefaciens*-mediated transformation with strain GV3101. To confirm targeted gene editing, the resulting plants were analyzed via HI-TOM sequencing at KMD Bioscience (Tianjing, China).

Then, we evaluated the phenotypes of the T_2_ transgenic generation by recording several key developmental traits. The flowering time was defined as the day the fifth flower opened post-sowing. At maturity, we measured silique length and seed count using pods sampled from the middle of the main inflorescence, while the 1000-seed weight was calculated from the total seed yield of the main inflorescence. Nine individual plants were measured for each line, with three biological replicates.

### 4.8. Plant Material, Culture Condition and qRT-PCR

Seeds of rapeseed ZS11 were provided by Southwest University, Chongqing, China (29°49′18″ N, 10°25′45″ E). All seeds were planted to a growth chamber using a peat-based soil mixture, Pindstrup 2 (Pindstrup, Ryomgaard, Denmark) with light 16 h at 22 °C, dark 8 h at 18 °C, and 60% relative humidity. The tissues of ZS11, including the root, middle stem, leaf, filament, and bud, were collected at the full-bloom stage, and seed and silique were collected at 14, 21 and 28 days after pollination (DAP) for RNA extraction. Moreover, the one-month-old leaves of ZS11 and transgenic rapeseed lines were also collected for RNA extraction. The drought treatment of ZS11 (one-month-old seedlings) was simulated by withholding irrigation for four days, while control plants (ZS11) were watered regularly (every two days). After undergoing treatment for four days, their leaves were collected for RNA extraction.

Total RNA was extracted from the samples using an RNAprep Pure Plant Kit (Tiangen, Beijing, China), and first-strand cDNA was synthesized using a HiScript III RT SuperMix for qPCR kit (Vazyme, Nanjing, China) according to the manufacturer’s instructions. Quantitative real-time (qRT)-PCR reactions were performed as described in the MIQE guidelines [47], with three technical replicates for each sample. Relative expression levels were performed using the 2^∆∆Ct^ method, with *Actin7* as the internal gene. All primers are listed in Table S7.

### 4.9. Statistical Analysis

GraphPad Prism 10 [48] was used for the statistical analysis, which involved a one-way analysis of variance and Student’s *t*-test, and results were presented as means ± standard errors.

## 5. Conclusions

Our study comprehensively explored the *ATXR* histone methyltransferase family in the rapeseed pan-genome. Our integrated analyses elucidated its evolutionary history within *Brassica* species, characterized its strong conservation and expansion mainly via whole-genome duplication, and revealed subfamily-specific expression patterns associated with plant development and stress responses. Crucially, functional validation identified *BnaATXR6* as a potential positive regulator of key yield-related traits in rapeseed. These findings provide valuable genetic resources and establish a solid foundation for exploiting epigenetic regulators in rapeseed breeding.

## Figures and Tables

**Table 1:** Copy-number variations of *BnaATXR* genes in 11 rapeseed accessions.

Ecotype	Winter				Semiwinter					Spring	
Accession	Darmor	Tapidor	Quinta	Express617	Gangan	Ningyou7	Shengli3	Zheyou7	ZS11	No2127	Westar
*ATXR1*	2	2	2	2	2	2	2	2	2	2	2
*ATXR2*	2	2	2	2	2	2	2	2	2	2	2
*ATXR3*	4	4	4	3	4	2	4	4	4	4	4
*ATXR4*	1	1	2	2	1	1	1	1	2	2	2
*ATXR5*	4	1	1	4	0	2	1	1	0	4	2
*ATXR6*	4	4	2	4	4	4	4	4	4	4	4
*ATXR7*	2	2	2	2	2	2	2	2	2	2	2
Total	19	16	15	19	15	15	16	16	16	20	18

## Data Availability

The original contributions presented in this study are included in the article/Supplementary Material. Further inquiries can be directed to the corresponding authors.

## Supplementary Materials

The following supporting information can be downloaded from https://www.mdpi.com/article/10.3390/plants15101458/s1: Table S1. The characteristics of ATXR family proteins in rapeseed; Table S2. The *ATXR* family genes in *B. rapa*; Table S3. The *ATXR* family genes in *B. oleracea*; Table S4. The *ATXR* family genes in *B. nigra*; Table S5. The *ATXR* family genes in *B. juncea*; Table S6. The *ATXR* family genes in *B. carinata*; Table S7. The sequences used for vector construction and qRT-RCR. Figure S1. The editing efficiency of *CR-BnaATXR6* lines plants; Figure S2. The diagram of 91 tissues in *B. napus *(ZS11).
