# Correction: Wu et al. Pan-Genomic Analysis and Functional Characterization of the *ATXR* Gene Family Highlights Its Role in Regulating Agronomic Traits in Rapeseed. *Plants* 2026, *15*, 1458

**DOI:** 10.3390/plants15162431

**Published:** 2026-08-10

**Authors:** Songze Wu, Minghao Zhang, Ruicheng Hu, Di Niu, Boyu Meng, Haikun Yang, Yuling Chen, Yonghai Fan, Kun Lu

**Affiliations:** 1 Integrative Science Center of Germplasm Creation in Western China (Chongqing) Science City, College of Agronomy and Biotechnology, Southwest University, Chongqing 400715, China; 17781201791@163.com (S.W.); 18311870271@163.com (M.Z.); 15681922709@163.com (R.H.); niudi0321@163.com (D.N.); beyul1340516624@email.swu.edu.cn (B.M.); haikun_2000@163.com (H.Y.); yulingchen1823@163.com (Y.C.); 2 Engineering Research Center of South Upland Agriculture, Ministry of Education, Chongqing 400715, China

In the original publication [1], there was a mistake in Figure 4 as published. The gene identifier *BnaC02.ATXR_ZS114* should be corrected to *BnaC02.ATXR4_ZS11*, *BnaC02.ATXR_ZS11* should be corrected to *BnaC02.ATXR6_ZS11*. The corrected Figure 4 appears below. The authors state that the scientific conclusions are unaffected. This correction was approved by the Academic Editor. The original publication has also been updated.

## Figures and Tables

**Figure 4:**
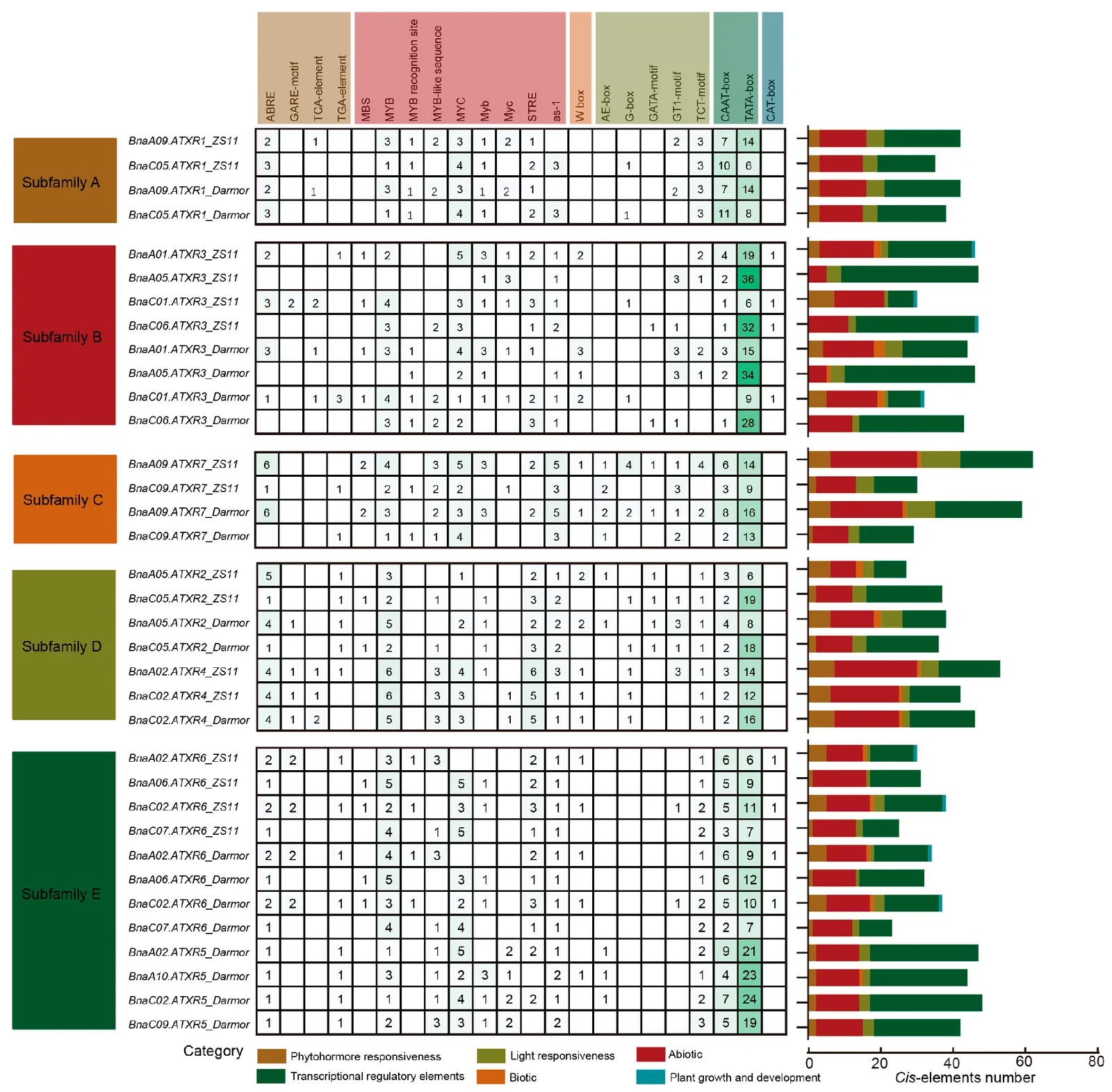
Summary of *cis*-acting elements in the promoter regions of *BnaATXR* genes in the ZS11 and Darmor genomes.
