# Supplementary material for: Pan-Genomic Analysis and Functional Characterization of the ATXR Gene Family Highlights Its Role in Regulating Agronomic Traits in Rapeseed
Source: Plants (Basel). 2026 May 10;15(10):1458. doi: 10.3390/plants15101458 (PMC13210918; doi:10.3390/plants15101458)
Supplement: Supplementary file 1 [file plants-15-01458-s001.zip › plants-4237138-supplementary.pdf]

Table S1 The characteristics of ATXR family proteins in rapeseed

Gene ID	Gene name	Amino Acid	Molecular Weight (Kda)	Isoelectric Point	Instability index	Aliphatic index	Average of Hydropathicity	mPLoc	Signal peptides	Transmembrane helixes
A09p40170_BnaDAR	*ATXR1*	541	60.62	6.16	42.63	85.77	-0.340	Nucleus	No signal	0
C05p24210_BnaDAR	*ATXR1*	541	60.55	6.97	43.43	85.21	-0.354	Nucleus	No signal	0
A05p26920_BnaDAR	*ATXR2*	436	48.48	4.80	50.98	81.49	-0.258	Nucleus	No signal	0
C05p41560_BnaDAR	*ATXR2*	471	52.32	4.84	53.76	83.50	-0.195	Nucleus	No signal	0
A01p22340_BnaDAR	*ATXR3*	2386	272.40	6.18	57.16	68.94	-0.799	Nucleus	No signal	0
A05p17060_BnaDAR	*ATXR3*	2396	273.25	6.20	53.30	67.93	-0.798	Nucleus	No signal	0
C01p27890_BnaDAR	*ATXR3*	2344	267.72	6.23	57.05	69.18	-0.787	Nucleus	No signal	0
C06p16490_BnaDAR	*ATXR3*	2395	273.01	6.15	54.88	68.80	-0.782	Nucleus	No signal	0
C02p02770_BnaDAR	*ATXR4*	329	36.30	6.96	53.18	81.61	-0.082	Mitochondrion	No signal	0
A02p00150_BnaDAR	*ATXR5*	391	44.13	8.42	53.98	77.26	-0.447	Chloroplast	No signal	0
A10p27660_BnaDAR	*ATXR5*	387	44.10	8.84	67.35	72.02	-0.636	Chloroplast	No signal	0
C02p04060_BnaDAR	*ATXR5*	392	44.19	8.45	57.73	78.55	-0.425	Chloroplast	No signal	0
C09p67720_BnaDAR	*ATXR5*	418	47.69	9.11	64.52	69.71	-0.660	Chloroplast	No signal	0
A02p40660_BnaDAR	*ATXR6*	345	39.12	9.08	53.04	77.68	-0.485	Nucleus	No signal	0
A06p35160_BnaDAR	*ATXR6*	345	39.05	8.83	52.12	77.97	-0.446	Nucleus	No signal	0
C02p60390_BnaDAR	*ATXR6*	525	59.67	8.70	47.15	80.38	-0.347	Nucleus	No signal	0
C07p43400_BnaDAR	*ATXR6*	345	39.11	8.75	45.74	78.81	-0.487	Nucleus	No signal	0
A09p19670_BnaDAR	*ATXR7*	1307	144.20	8.59	49.24	75.81	-0.518	Nucleus	No signal	0
C09p25030_BnaDAR	*ATXR7*	1314	145.52	8.39	50.05	73.71	-0.573	Nucleus	No signal	0
A09p031240_BnaEXP	*ATXR1*	540	60.52	5.92	43.86	85.57	-0.355	Nucleus	No signal	0
C05p020650_BnaEXP	*ATXR1*	541	60.55	6.97	43.43	85.21	-0.354	Nucleus	No signal	0
A05p021340_BnaEXP	*ATXR2*	436	48.51	4.80	51.11	80.83	-0.263	Nucleus	No signal	0
C05p028200_BnaEXP	*ATXR2*	494	55.56	4.90	51.77	77.79	-0.354	Nucleus	No signal	0
A01p022250_BnaEXP	*ATXR3*	2386	272.40	6.18	57.16	68.94	-0.799	Nucleus	No signal	0
C01p020380_BnaEXP	*ATXR3*	2425	277.31	6.36	56.45	70.56	-0.744	Cytoplasm	No signal	2
C06p012380_BnaEXP	*ATXR3*	2357	268.68	6.29	54.74	69.04	-0.781	Nucleus	No signal	0
A02p001810_BnaEXP	*ATXR4*	329	36.43	7.45	52.31	82.19	-0.074	Chloroplast	No signal	0
C02p002150_BnaEXP	*ATXR4*	329	36.30	6.96	53.18	81.61	-0.082	Mitochondrion	No signal	0
A02p002840_BnaEXP	*ATXR5*	393	44.22	8.47	55.90	77.10	-0.472	Chloroplast	No signal	0
A10p023720_BnaEXP	*ATXR5*	387	44.10	8.84	67.35	72.02	-0.636	Chloroplast	No signal	0
C02p003320_BnaEXP	*ATXR5*	390	43.96	8.48	57.14	78.95	-0.439	Chloroplast	No signal	0
C09p051840_BnaEXP	*ATXR5*	385	44.02	8.91	66.17	70.10	-0.669	Chloroplast	No signal	0
A02p037690_BnaEXP	*ATXR6*	345	39.12	9.08	53.04	77.68	-0.485	Nucleus	No signal	0
A06p027850_BnaEXP	*ATXR6*	345	38.96	8.91	49.45	79.39	-0.419	Nucleus	No signal	0
C02p038120_BnaEXP	*ATXR6*	342	38.91	9.20	55.74	75.79	-0.508	Nucleus	No signal	0
A09p016750_BnaEXP	*ATXR7*	1288	141.97	8.50	49.61	73.75	-0.562	Nucleus	No signal	0
C09p020030_BnaEXP	*ATXR7*	1261	139.53	8.52	48.60	73.47	-0.582	Nucleus	No signal	0
BnaA09T0408700GG	*ATXR1*	541	60.62	6.16	42.63	85.77	-0.340	Nucleus	No signal	0

BnaC05T0210600GG	*ATXR1*	541	60.55	6.97	43.43	85.21	-0.354	Nucleus	No signal	0
BnaA05T0237300GG	*ATXR2*	436	48.51	4.80	51.11	80.83	-0.263	Nucleus	No signal	0
BnaC05T0361400GG	*ATXR2*	471	52.32	4.84	53.76	83.50	-0.195	Nucleus	No signal	0
BnaA01T0192800GG	*ATXR3*	2386	272.40	6.19	57.16	68.94	-0.799	Nucleus	No signal	0
BnaA05T0136300GG	*ATXR3*	2396	273.36	6.23	54.32	67.64	-0.806	Nucleus	No signal	0
BnaC01T0232300GG	*ATXR3*	2344	267.70	6.21	56.80	69.18	-0.784	Nucleus	No signal	0
BnaC06T0131800GG	*ATXR3*	2395	273.01	6.15	54.88	68.80	-0.782	Nucleus	No signal	0
BnaA02T0019300GG	*ATXR4*	329	36.38	7.03	52.49	83.68	-0.073	Mitochondrion	No signal	0
BnaA02T0367300GG	*ATXR6*	269	30.73	9.42	48.96	77.17	-0.575	Nucleus	No signal	0
BnaA06T0315500GG	*ATXR6*	370	41.76	8.86	47.78	85.59	-0.311	Nucleus	No signal	0
BnaC02T0475600GG	*ATXR6*	342	38.91	9.20	55.74	75.79	-0.508	Nucleus	No signal	0
BnaC07T0353600GG	*ATXR6*	345	39.11	8.75	45.74	78.81	-0.487	Nucleus	No signal	0
BnaA09T0181600GG	*ATXR7*	1282	141.45	8.34	50.02	74.86	-0.550	Nucleus	No signal	0
BnaC03T0412600GG	*ATXR7*	1282	141.55	8.23	48.87	73.34	-0.582	Nucleus	No signal	0
chrA09g003878	*ATXR1*	540	60.46	5.97	41.58	86.46	-0.333	Nucleus	No signal	0
chrC05g002186	*ATXR1*	541	60.55	6.97	43.43	85.21	-0.354	Nucleus	No signal	0
chrA05g002263	*ATXR2*	450	49.95	4.78	51.84	83.73	-0.222	Chloroplast	No signal	0
chrC05g003715	*ATXR2*	469	52.08	4.87	55.91	83.03	-0.209	Nucleus	No signal	0
chrA05g001495	*ATXR3*	2396	273.22	6.20	53.24	67.93	-0.798	Nucleus	No signal	0
Scaffold128g000005	*ATXR3*	2386	272.29	6.20	57.52	69.06	-0.796	Nucleus	No signal	0
chrA02g000279	*ATXR4*	329	36.36	7.44	52.66	81.61	-0.094	Chloroplast	No signal	0
chrA02g000410	*ATXR5*	390	43.95	8.45	54.35	77.21	-0.467	Chloroplast	No signal	0
chrA10g002664	*ATXR5*	383	43.68	8.91	64.87	72.77	-0.626	Chloroplast	No signal	0
chrA02g004805	*ATXR6*	550	62.07	8.97	47.14	78.18	-0.404	Nucleus	No signal	0
chrA06g002901	*ATXR6*	345	39.08	8.75	50.62	77.39	-0.463	Nucleus	No signal	0
chrC02g004061	*ATXR6*	342	38.75	9.06	54.16	76.37	-0.483	Nucleus	No signal	0
chrC07g003808	*ATXR6*	345	39.11	8.75	45.74	78.81	-0.487	Nucleus	No signal	0
chrA09g002074	*ATXR7*	1282	141.17	8.46	49.71	74.02	-0.556	Nucleus	No signal	0
chrC09g002105	*ATXR7*	1288	142.18	8.28	48.62	73.30	-0.582	Nucleus	No signal	0
BnaA09T0375200NO	*ATXR1*	541	60.53	6.16	42.71	85.58	-0.334	Nucleus	No signal	0
BnaC05T0211700NO	*ATXR1*	541	60.55	6.97	43.43	85.21	-0.354	Nucleus	No signal	0
BnaA05T0258800NO	*ATXR2*	450	49.91	4.78	51.71	84.38	-0.216	Chloroplast	No signal	0
BnaC05T0356700NO	*ATXR2*	471	52.32	4.84	53.76	83.50	-0.195	Nucleus	No signal	0
BnaA01T0165200NO	*ATXR3*	2386	272.52	6.20	56.80	68.89	-0.803	Nucleus	No signal	0
BnaA05T0083900NO	*ATXR3*	2396	273.36	6.23	54.32	67.64	-0.806	Nucleus	No signal	0
BnaC01T0145600NO	*ATXR3*	2344	267.72	6.23	57.05	69.18	-0.787	Nucleus	No signal	0
BnaC06T0134300NO	*ATXR3*	2395	273.01	6.15	54.88	68.80	-0.782	Nucleus	No signal	0
BnaA02T0022100NO	*ATXR4*	329	36.43	7.45	52.31	82.19	-0.074	Chloroplast	No signal	0
Bnascaffold1876T0035100NO	*ATXR4*	329	36.32	6.96	53.18	81.00	-0.089	Mitochondrion	No signal	0
BnaA02T0033200NO	*ATXR5*	393	44.21	8.45	55.49	76.62	-0.470	Chloroplast	No signal	0
BnaA10T0190000NO	*ATXR5*	387	44.10	8.84	67.35	72.02	-0.636	Chloroplast	No signal	0
BnaC09T0496400NO	*ATXR5*	385	44.02	8.91	66.17	70.10	-0.669	Chloroplast	No signal	0
Bnascaffold1876T0023600N	*ATXR5*	390	44.03	8.34	57.17	78.21	-0.462	Chloroplast	No signal	0

O										
BnaA02T0377500NO	*ATXR6*	345	39.11	9.08	53.20	76.55	-0.501	Nucleus	No signal	0
BnaA06T0329500NO	*ATXR6*	345	38.94	8.83	49.21	79.10	-0.445	Nucleus	No signal	0
BnaC02T0465300NO	*ATXR6*	342	38.91	9.20	55.74	75.79	-0.508	Nucleus	No signal	0
BnaC07T0350000NO	*ATXR6*	345	39.11	8.75	45.74	78.81	-0.487	Nucleus	No signal	0
BnaA09T0120800NO	*ATXR7*	1332	147.48	8.55	50.02	77.24	-0.502	Nucleus	No signal	0
BnaC09T0172700NO	*ATXR7*	1313	145.44	8.39	50.56	73.47	-0.577	Nucleus	No signal	0
BnaA09T0420700QU	*ATXR1*	540	60.52	5.92	43.86	85.57	-0.355	Nucleus	No signal	0
BnaC05T0175600QU	*ATXR1*	541	60.55	6.97	43.43	85.21	-0.354	Nucleus	No signal	0
BnaA05T0243000QU	*ATXR2*	436	48.48	4.80	50.98	81.49	-0.258	Nucleus	No signal	0
BnaC05T0310500QU	*ATXR2*	469	52.14	4.87	55.38	83.03	-0.201	Nucleus	No signal	0
BnaA01T0145200QU	*ATXR3*	2386	272.47	6.21	57.44	68.77	-0.804	Nucleus	No signal	0
BnaA05T0145100QU	*ATXR3*	2396	273.25	6.20	53.30	67.93	-0.798	Nucleus	No signal	0
BnaC01T0161300QU	*ATXR3*	2344	267.72	6.23	57.05	69.18	-0.787	Nucleus	No signal	0
BnaC06T0132700QU	*ATXR3*	2395	273.01	6.15	54.88	68.80	-0.782	Nucleus	No signal	0
BnaA02T0018900QU	*ATXR4*	329	36.43	7.45	52.31	52.31	-0.074	Chloroplast	No signal	0
BnaC08T0291000QU	*ATXR4*	329	36.32	6.96	53.18	81.00	-0.089	Mitochondrion	No signal	0
BnaC09T0523500QU	*ATXR5*	417	47.56	9.16	63.87	69.88	-0.653	Chloroplast	No signal	0
BnaC02T0483400QU	*ATXR6*	342	38.91	9.20	55.74	75.79	-0.508	Nucleus	No signal	0
BnaC07T0345900QU	*ATXR6*	275	31.56	8.61	38.24	75.85	-0.649	Nucleus	No signal	0
BnaA09T0175500QU	*ATXR7*	1281	141.07	8.46	49.81	73.85	-0.559	Nucleus	No signal	0
BnaC09T0200800QU	*ATXR7*	1287	142.09	8.28	48.72	73.13	-0.585	Nucleus	No signal	0
BnaA09T0445900SL	*ATXR1*	540	60.38	5.86	42.74	85.20	-0.343	Nucleus	No signal	0
BnaC05T0236200SL	*ATXR1*	541	60.55	6.97	43.43	85.21	-0.354	Nucleus	No signal	0
BnaA05T0275900SL	*ATXR2*	450	49.95	4.78	51.84	83.73	-0.222	Chloroplast	No signal	0
BnaC05T0367400SL	*ATXR2*	471	52.32	4.84	53.76	83.50	-0.195	Nucleus	No signal	0
BnaA01T0170600SL	*ATXR3*	2386	272.40	6.18	57.16	68.94	-0.799	Nucleus	No signal	0
BnaA07T0044900SL	*ATXR3*	2396	273.31	6.20	53.16	67.93	-0.797	Nucleus	No signal	0
BnaC01T0224600SL	*ATXR3*	2344	267.70	6.21	56.80	69.18	-0.784	Nucleus	No signal	0
BnaC06T0130200SL	*ATXR3*	2395	273.01	6.15	54.88	68.80	-0.782	Nucleus	No signal	0
BnaA02T0019900SL	*ATXR4*	329	36.38	7.03	52.49	83.68	-0.073	Mitochondrion	No signal	0
BnaA10T0211000SL	*ATXR5*	383	43.68	8.91	64.87	72.77	-0.626	Chloroplast	No signal	0
BnaA02T0375700SL	*ATXR6*	345	39.20	9.08	53.85	78.52	-0.472	Nucleus	No signal	0
BnaA06T0314300SL	*ATXR6*	370	41.86	9.00	48.88	85.32	-0.303	Nucleus	No signal	0
BnaC02T0396800SL	*ATXR6*	342	38.75	9.06	54.16	76.37	-0.483	Nucleus	No signal	0
BnaC07T0328700SL	*ATXR6*	222	25.37	6.45	36.78	80.36	-0.530	Nucleus	No signal	0
BnaA09T0229700SL	*ATXR7*	1312	144.92	8.62	48.43	75.52	-0.517	Chloroplast	No signal	0
BnaC09T0058700SL	*ATXR7*	1319	146.14	8.39	50.08	73.65	-0.567	Nucleus	No signal	0
BnaA09T0345900TA	*ATXR1*	540	60.52	5.92	43.86	85.57	-0.355	Nucleus	No signal	0
BnaC05T0213100TA	*ATXR1*	541	60.55	6.97	43.43	85.21	-0.354	Nucleus	No signal	0
BnaA05T0292200TA	*ATXR2*	450	49.91	4.78	51.71	84.38	-0.216	Chloroplast	No signal	0
BnaC05T0347900TA	*ATXR2*	471	52.32	4.84	53.76	83.50	-0.195	Nucleus	No signal	0
BnaA01T0121000TA	*ATXR3*	2386	272.40	6.18	57.16	68.94	-0.799	Nucleus	No signal	0
BnaA05T0140300TA	*ATXR3*	2396	273.25	6.20	53.30	67.93	-0.798	Nucleus	No signal	0

BnaC01T0182900TA	*ATXR3*	2344	267.72	6.23	57.05	69.18	-0.787	Nucleus	No signal	0
BnaC06T0130200TA	*ATXR3*	2395	273.01	6.15	54.88	68.80	-0.782	Nucleus	No signal	0
BnaC02T0023000TA	*ATXR4*	329	36.30	6.96	53.18	81.61	-0.082	Mitochondrion	No signal	0
BnaC09T0539800TA	*ATXR5*	385	44.02	8.91	66.17	70.10	-0.669	Chloroplast	No signal	0
BnaA02T0342700TA	*ATXR6*	249	28.41	9.21	39.89	78.67	-0.539	Nucleus	No signal	0
BnaA06T0301500TA	*ATXR6*	345	38.99	8.91	49.45	79.10	-0.426	Nucleus	No signal	0
BnaC02T0409300TA	*ATXR6*	525	59.67	8.70	47.15	80.38	-0.347	Nucleus	No signal	0
BnaC07T0354700TA	*ATXR6*	346	39.16	8.59	48.43	77.75	-0.515	Nucleus	No signal	0
BnaA09T0167100TA	*ATXR7*	1306	144.11	8.59	49.48	75.57	-0.522	Nucleus	No signal	0
BnaC09T0209400TA	*ATXR7*	1281	141.42	8.39	48.44	73.55	-0.577	Nucleus	No signal	0
BnaA09T0414400WE	*ATXR1*	541	60.53	6.16	42.71	85.58	-0.334	Nucleus	No signal	0
BnaC05T0210200WE	*ATXR1*	541	60.55	6.97	43.43	85.21	-0.354	Nucleus	No signal	0
BnaA05T0292100WE	*ATXR2*	436	48.48	4.80	50.98	81.49	-0.258	Nucleus	No signal	0
BnaC05T0345500WE	*ATXR2*	469	52.08	4.87	55.91	83.03	-0.209	Nucleus	No signal	0
BnaA01T0117800WE	*ATXR3*	2386	272.40	6.18	57.16	68.94	-0.799	Nucleus	No signal	0
BnaA05T0149200WE	*ATXR3*	2396	273.25	6.20	53.30	67.93	-0.798	Nucleus	No signal	0
BnaC01T0196100WE	*ATXR3*	2344	267.72	6.23	57.05	69.18	-0.787	Nucleus	No signal	0
BnaC06T0128500WE	*ATXR3*	2395	273.01	6.15	54.88	68.80	-0.782	Nucleus	No signal	0
BnaA02T0022900WE	*ATXR4*	329	36.36	7.44	52.66	81.61	-0.094	Chloroplast	No signal	0
BnaC02T0019100WE	*ATXR4*	329	36.32	6.96	53.18	81.00	-0.089	Mitochondrion	No signal	0
BnaC02T0030900WE	*ATXR5*	392	44.19	8.45	57.73	78.55	-0.425	Chloroplast	No signal	0
BnaC09T0532900WE	*ATXR5*	384	43.89	8.97	65.47	70.29	-0.661	Chloroplast	No signal	0
BnaA02T0381600WE	*ATXR6*	269	30.72	9.42	50.24	77.17	-0.581	Nucleus	No signal	0
BnaA06T0359900WE	*ATXR6*	305	34.75	9.35	44.24	88.52	-0.345	Cytoplasm	No signal	0
BnaC02T0498900WE	*ATXR6*	342	38.80	9.00	54.66	76.37	-0.492	Nucleus	No signal	0
BnaC07T0359600WE	*ATXR6*	275	31.57	8.61	37.61	77.27	-0.629	Nucleus	No signal	0
BnaA09T0174700WE	*ATXR7*	1282	141.45	8.34	50.02	74.86	-0.550	Nucleus	No signal	0
BnaC09T0218500WE	*ATXR7*	1313	145.13	8.33	48.85	74.06	-0.571	Nucleus	No signal	0
BnaA09T0394400ZY	*ATXR1*	540	60.46	5.97	41.58	86.46	-0.333	Nucleus	No signal	0
BnaC05T0210000ZY	*ATXR1*	541	60.55	6.97	43.43	85.21	-0.354	Nucleus	No signal	0
BnaA05T0300300ZY	*ATXR2*	436	48.51	4.80	51.11	80.83	-0.263	Nucleus	No signal	0
BnaC05T0372800ZY	*ATXR2*	469	52.08	4.87	55.91	83.03	-0.209	Nucleus	No signal	0
BnaA01T0197300ZY	*ATXR3*	2386	272.40	6.18	57.16	68.94	-0.799	Nucleus	No signal	0
BnaA05T0145700ZY	*ATXR3*	2396	273.26	6.21	53.50	67.93	-0.798	Nucleus	No signal	0
BnaC06T0133300ZY	*ATXR3*	2395	273.01	6.15	54.88	68.80	-0.782	Nucleus	No signal	0
BnaC09T0424800ZY	*ATXR3*	2344	267.70	6.21	56.80	69.18	-0.784	Nucleus	No signal	0
BnaA02T0019600ZY	*ATXR4*	329	36.38	7.03	52.49	83.68	-0.073	Mitochondrion	No signal	0
BnaA02T0029600ZY	*ATXR5*	390	43.95	8.45	54.35	77.21	-0.467	Chloroplast	No signal	0
BnaA02T0401600ZY	*ATXR6*	345	39.17	9.14	52.37	76.55	-0.504	Nucleus	No signal	0
BnaA06T0322200ZY	*ATXR6*	305	34.75	9.35	44.24	88.52	-0.345	Cytoplasm	No signal	0
BnaC02T0353100ZY	*ATXR6*	342	38.75	9.06	54.16	76.37	-0.483	Nucleus	No signal	0
BnaC07T0340600ZY	*ATXR6*	345	39.11	8.75	45.74	78.81	-0.487	Nucleus	No signal	0
BnaA09T0180900ZY	*ATXR7*	1312	144.74	8.61	49.24	75.52	-0.522	Nucleus	No signal	0
BnaC09T0190900ZY	*ATXR7*	1319	146.07	8.42	50.31	73.43	-0.577	Nucleus	No signal	0

BnaA09T0443600ZS	*ATXR1*	541	60.62	6.16	42.63	85.77	-0.340	Nucleus	No signal	0
BnaC05T0219300ZS	*ATXR1*	541	60.55	6.97	43.43	85.21	-0.354	Nucleus	No signal	0
BnaA05T0340500ZS	*ATXR2*	450	49.91	4.78	51.71	84.38	-0.216	Chloroplast	No signal	0
BnaC05T0364500ZS	*ATXR2*	469	52.08	4.87	55.91	83.03	-0.209	Nucleus	No signal	0
BnaA01T0198400ZS	*ATXR3*	2386	272.37	6.16	57.10	68.94	-0.800	Nucleus	No signal	0
BnaA05T0150300ZS	*ATXR3*	2396	273.25	6.20	53.30	67.93	-0.798	Nucleus	No signal	0
BnaC01T0250400ZS	*ATXR3*	1794	204.28	5.62	53.48	76.76	-0.564	Nucleus	No signal	0
BnaC06T0144300ZS	*ATXR3*	2395	273.01	6.15	54.88	68.80	-0.782	Nucleus	No signal	0
BnaA02T0023000ZS	*ATXR4*	329	36.38	7.03	52.49	83.68	-0.073	Mitochondrion	No signal	0
BnaC02T0024700ZS	*ATXR4*	329	36.32	6.96	53.18	81.00	-0.089	Mitochondrion	No signal	0
BnaA02T0386700ZS	*ATXR6*	269	30.73	9.42	48.96	77.17	-0.575	Nucleus	No signal	0
BnaA06T0328100ZS	*ATXR6*	305	34.75	9.35	44.24	88.52	-0.345	Cytoplasm	No signal	0
BnaC02T0516800ZS	*ATXR6*	525	59.67	8.70	47.15	80.38	-0.347	Nucleus	No signal	0
BnaC07T0366000ZS	*ATXR6*	269	30.79	8.76	40.47	78.62	-0.575	Mitochondrion	No signal	0
BnaA09T0185500ZS	*ATXR7*	1282	141.17	8.46	49.71	74.02	-0.556	Nucleus	No signal	0
BnaC09T0211700ZS	*ATXR7*	1314	145.39	8.38	49.84	73.63	-0.573	Nucleus	No signal	0

Table S2 The ATXR family genes in *B. rapa*

Chiifu		Z1		BRO		ECD04	
Gene ID	Gene name	Gene ID	Gene name	Gene ID	Gene name	Gene ID	Gene name
BraA09g041470.4.1C.1	*ATXR1*	*A09p51940.2_BraZ1*	*ATXR1*	*A09p38840.1_BraBRO*	*ATXR1*	*BraA09t004502E*	*ATXR1*
BraA05g027360.4.1C.1	*ATXR2*	*A05p32810.2_BraZ1*	*ATXR2*	*A05p24460.1_BraBRO*	*ATXR2*	*BraA05t017080E*	*ATXR2*
BraA01g021640.4.1C.1	*ATXR3*	*A01p22750.2_BraZ1*	*ATXR3*	*A01p21390.1_BraBRO*	*ATXR3*	*BraA01t025859E*	*ATXR3*
BraA05g016500.4.1C.1	*ATXR3*	*A05p17630.2_BraZ1*	*ATXR3*	*A05p15740.1_BraBRO*	*ATXR3*	*BraA05t015505E*	*ATXR3*
BraA02g002100.4.1C.1	*ATXR4*	*A02p02310.2_BraZ1*	*ATXR4*	*A02p02150.1_BraBRO*	*ATXR4*	*BraA02t028696E*	*ATXR4*
BraA02g003210.4.1C.1	*ATXR5*	*A02p03520.2_BraZ1*	*ATXR5*	*A02p03270.1_BraBRO*	*ATXR5*	*BraA02t028818E*	*ATXR5*
BraA06g034410.4.1C.1	*ATXR6*	*A10p28740.2_BraZ1*	*ATXR5*	*A10p27650.1_BraBRO*	*ATXR5*	*BraA10t047501E*	*ATXR5*
BraA09g020170.4.1C.1	*ATXR7*	*A02p48890.2_BraZ1*	*ATXR6*	*A06p31740.1_BraBRO*	*ATXR6*	*BraA02t032902E*	*ATXR6*
		A06p42520.2_BraZ1	*ATXR6*	*A09p19370.1_BraBRO*	*ATXR7*	*BraA06t022527E*	*ATXR6*
		A09p21210.2_BraZ1	*ATXR7*			*BraA09t001920E*	*ATXR7*

Table S3 The ATXR family genes in *B. oleracea*

TO1000		HDEM		Korso		OX-heart	
Gene ID	Gene name	Gene ID	Gene name	Gene ID	Gene name	Gene ID	Gene name
Bo5g045060.1	*ATXR1*	*BolC5t31157H*	*ATXR1*	*BolK_5g23080.1*	*ATXR1*	*BolO_5g24600.1*	*ATXR1*

Bo5g098680.1	*ATXR2*	*BolC5t32893H*	*ATXR2*	*BolK_5g40600.1*	*ATXR2*	*BolO_5g42800.1*	*ATXR2*
Bo1g057090.1	*ATXR3*	*BolC1t02722H*	*ATXR3*	*BolK_1g27570.1*	*ATXR3*	*BolO_1g27710.1*	*ATXR3*
Bo6g053660.1	*ATXR3*	*BolC6t36852H*	*ATXR3*	*BolK_6g16470.1*	*ATXR3*	*BolO_6g16520.1*	*ATXR3*
Bo2g007290.1	*ATXR4*	*BolC2t06242H*	*ATXR4*	*BolK_2g02700.1*	*ATXR4*	*BolO_2g02810.1*	*ATXR4*
Bo2g009530.1	*ATXR5*	*BolC2t06368H*	*ATXR5*	*BolK_2g03940.1*	*ATXR5*	*BolO_2g04060.1*	*ATXR5*
Bo9g173800.1	*ATXR5*	*BolC9t59534H*	*ATXR5*	*BolK_9g64160.1*	*ATXR5*	*BolO_9g65990.1*	*ATXR5*
Bo2g159500.1	*ATXR6*	*BolC9t59536H*	*ATXR5*	*BolK_9g64180.1*	*ATXR5*	*BolO_2g62280.1*	*ATXR6*
Bo7g096970.1	*ATXR6*	*BolC2t12094H*	*ATXR6*	*BolK_2g59710.1*	*ATXR6*	*BolO_7g44990.1*	*ATXR6*
Bo9g058280.1	*ATXR7*	*BolC7t44867H*	*ATXR6*	*BolK_7g43640.1*	*ATXR6*	*BolO_9g24470.1*	*ATXR7*
		BolC9t55397H	*ATXR7*	*BolK_9g23510.1*	*ATXR7*		

Table S4 The *ATXR* family genes in *B. nigra*

CN115125		NI100	
Gene ID	Gene name	Gene ID	Gene name
BniB04g040040.2N.1	*ATXR1*	*BniB04g043620.1C2.1*	*ATXR1*
BniB01g041200.2N.1	*ATXR2*	*BniB01g043670.1C2.1*	*ATXR2*
BniB05g020990.2N.1	*ATXR3*	*BniB05g021440.1C2.1*	*ATXR3*
BniB06g058470.2N.1	*ATXR3*	*BniB06g064890.1C2.1*	*ATXR3*
BniB05g028720.2N.1	*ATXR4*	*BniB05g028860.1C2.1*	*ATXR4*
BniB02g052050.2N.1	*ATXR5*	*BniB02g056280.1C2.1*	*ATXR5*
BniB05g027130.2N.1	*ATXR5*	*BniB05g027220.1C2.1*	*ATXR5*
BniB02g077240.2N.1	*ATXR6*	*BniB02g083820.1C2.1*	*ATXR6*
BniB04g018300.2N.1	*ATXR7*	*BniB04g019740.1C2.1*	*ATXR7*

Table S5 The ATXR family genes in *B. juncea*

AU213		Tumida	
Gene ID	Gene name	Gene ID	Gene name
BjuOA09G41500	*ATXR1*	*BjuVA09G40560*	*ATXR1*
BjuOB04G37410	*ATXR1*	*BjuVB04G36170*	*ATXR1*
BjuOA05G28040	*ATXR2*	*BjuVA05G27010*	*ATXR2*
BjuOB01G34570	*ATXR2*	*BjuVB01G32520*	*ATXR2*
BjuOA01G22930	*ATXR3*	*BjuVA01G22860*	*ATXR3*
BjuOA05G16780	*ATXR3*	*BjuVA05G16630*	*ATXR3*
BjuOB05G18270	*ATXR3*	*BjuVB05G20580*	*ATXR3*
BjuOB06G50170	*ATXR3*	*BjuVB06G49860*	*ATXR3*
BjuOA02G02380	*ATXR4*	*BjuVA02G02300*	*ATXR4*
BjuOB05G25380	*ATXR4*	*BjuVB05G27360*	*ATXR4*
BjuOA02G03560	*ATXR5*	*BjuVA02G03450*	*ATXR5*
BjuOA10G28490	*ATXR5*	*BjuVA10G28080*	*ATXR5*
BjuOB02G42420	*ATXR5*	*BjuVB02G39940*	*ATXR5*
BjuOB05G23910	*ATXR5*	*BjuVB05G25950*	*ATXR5*

BjuOA02G46410	*ATXR6*	*BjuVA02G45880*	*ATXR6*
BjuOA06G31680	*ATXR6*	*BjuVA06G34070*	*ATXR6*
BjuOB02G67570	*ATXR6*	*BjuVB02G63660*	*ATXR6*
BjuOA09G20440	*ATXR7*	*BjuVA09G19740*	*ATXR7*
BjuOB04G17320	*ATXR7*	*BjuVB04G17100*	*ATXR7*

Table S6 The ATXR family genes in *B. carinata*

10167		zd-1	
Gene ID	Gene name	Gene ID	Gene name
Bca101B4G019620.1	*ATXR1*	*BcaC05g26390*	*ATXR1*
Bca101C5G007700.1	*ATXR1*	*BcaB06g26287*	*ATXR2*
Bca101B1G024310.1	*ATXR2*	*BcaC05g28122*	*ATXR2*
Bca101C5G028670.1	*ATXR2*	*BcaB02g07835*	*ATXR3*
Bca101B5G002590.1	*ATXR3*	*BcaC08g44245*	*ATXR3*
Bca101B6G039990.1	*ATXR3*	*BcaC09g50202*	*ATXR3*
Bca101C1G012260.1	*ATXR3*	*BcaNung00300*	*ATXR3*
Bca101C6G007370.1	*ATXR3*	*BcaB05g21660*	*ATXR4*
Bca101B5G009340.1	*ATXR4*	*BcaB05g21529*	*ATXR5*
Bca101C2G008940.1	*ATXR4*	*BcaB08g37166*	*ATXR5*
Bca101B2G033670.1	*ATXR5*	*BcaB08g37181*	*ATXR5*
Bca101B5G007760.1	*ATXR5*	*BcaC03g13275*	*ATXR5*
Bca101C2G015300.1	*ATXR5*	*BcaNung01225*	*ATXR6*
Bca101C9G053480.1	*ATXR5*	*BcaNung01226*	*ATXR6*
Bca101B2G059350.1	*ATXR6*	*BcaB07g31077*	*ATXR7*
Bca101C7G034430.1	*ATXR6*	*BcaC04g21996*	*ATXR7*
Bca101B4G051460.1	*ATXR7*		
Bca101C9G009950.1	*ATXR7*		

Table S7 The sequences used for vector construction and qRT-RCR

Name	5′-3′
sgRNA1	AGGAAAAGGACAAGCTTGGTGA
sgRNA2	TATGAAGGGTTCACAGTAGAG
qBnaA09.ATXR1-F	ACTGTTGCAGAGCCTCAGATCC
qBnaA09.ATXR1-R	ACCCACTTAGGATCCAATCAGAAA
qBnaA05.ATXR2-F	TTCAATCAGCTAAGAAGAAGATGCA
qBnaA05.ATXR2-R	GCGCATGATCCACTGCAGTAA
qBnaA01.ATXR3-F	GCCTCTACTGAATATCATGGACAAG
qBnaA01.ATXR3-R	CACCCTCTTCCACTTCATCCTT
qBnaA02.ATXR4-F	AACCTCTGCCTCATCTGAATCAC
qBnaA02.ATXR4-R	GAAGTTGTGCGTCCTACAATAATCA
qBnaC02.ATXR4-F	CCTTAAACCTCTGTCTCATCTGAACA
qBnaC02.ATXR4-R	GGTACTTGAAGTTGTGCGTCCT
qBnaC07.ATXR6-F	CCTCTTGTTCAGACCAAACTAATCG
qBnaC07.ATXR6-R	CCTTGCCAGATACATAAGTGAGCT

ATXR6p:GUS-F	AAGACCTACCAGAACTCCCTGTTG
ATXR6p:GUS-R	ATTGCAATCTCACCTCCTTCAAT

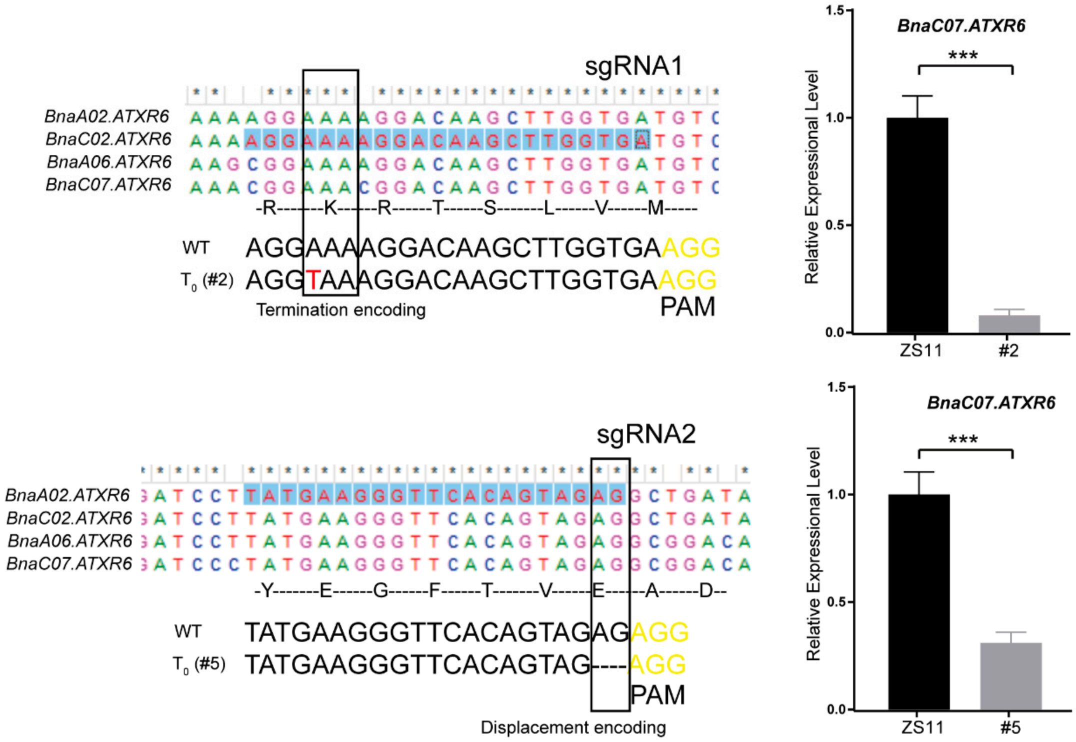

Figure S1. The editing efficiency of *CR-BnaATXR6* lines plants. * $P < 0.05$, ** $P < 0.01$, Student's *t*-test.

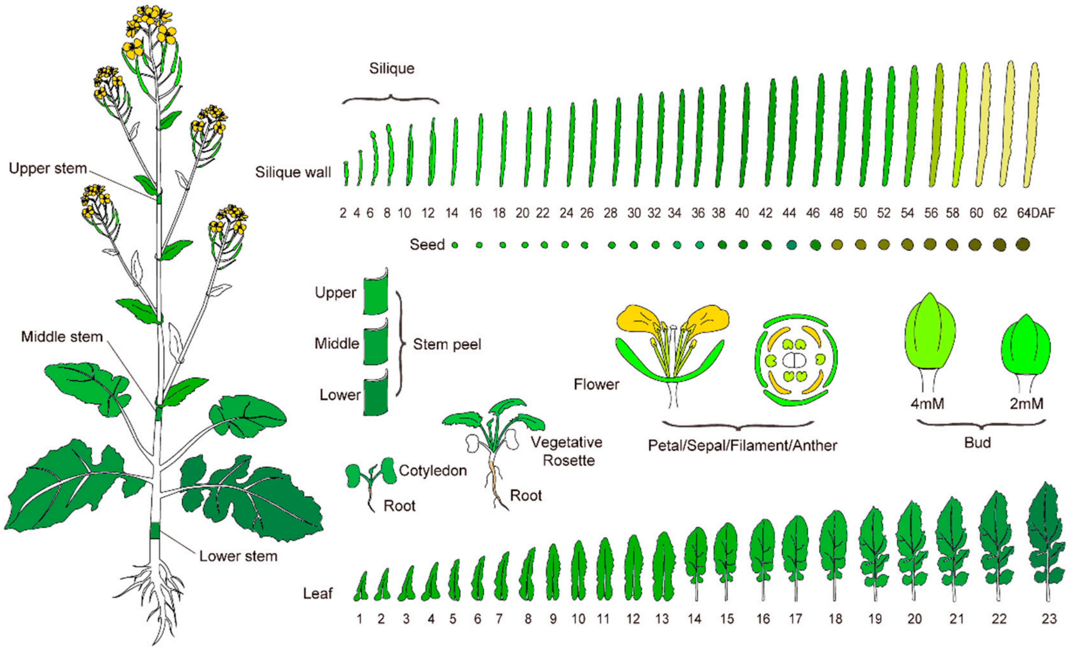

Figure S2. The diagram of 91 tissues in *B. napus* (ZS11). The Figure downloaded from BnTIR database
